# An integrated approach to improve clinical trial efficiency: Linking a clinical trial management system into the Research Integrated Network of Systems

**DOI:** 10.1017/cts.2022.382

**Published:** 2022-04-01

**Authors:** Royce Sampson, Steve Shapiro, Wenjun He, Signe Denmark, Katie Kirchoff, Kyle Hutson, Rechelle Paranal, Leila Forney, Kimberly McGhee, Jillian Harvey

**Affiliations:** 1 South Carolina Clinical & Translational Research Institute, Medical University of South Carolina, Charleston, SC, USA; 2 Office of Clinical Research, Office of the Vice President for Research, Medical University of South Carolina, Charleston, SC, USA; 3 Department of Psychiatry and Behavioral Sciences, Medical University of South Carolina, Charleston, SC, USA; 4 Biomedical Informatics Center, Medical University of South Carolina, Charleston, SC, USA; 5 Academic Affairs Faculty, Medical University of South Carolina, Charleston, SC, USA; 6 Department of Healthcare Leadership and Management, Medical University of South Carolina, Charleston, SC, USA

**Keywords:** Clinical trial management system, data integration, clinical and translational science, clinical trial, clinical trial efficiency, clinical trial metrics, accrual, low-accruing trials, patient accrual, financial performance, enrollment, cost-effective

## Abstract

Low-accruing clinical trials delay translation of research breakthroughs into the clinic, expose participants to risk without providing meaningful clinical insight, increase the cost of therapies, and waste limited resources. By tracking patient accrual, Clinical and Translational Science Awards hubs can identify at-risk studies and provide them the support needed to reach recruitment goals and maintain financial solvency. However, tracking accrual has proved challenging because relevant patient- and protocol-level data often reside in siloed systems. To address this fragmentation, in September 2020 the South Carolina Clinical and Translational Research Institute, with an academic home at the Medical University of South Carolina, implemented a clinical trial management system (CTMS), with its access to patient-level data, and incorporated it into its Research Integrated Network of Systems (RINS), which links study-level data across disparate systems relevant to clinical research. Within the first year of CTMS implementation, 324 protocols were funneled through CTMS/RINS, with more than 2600 participants enrolled. Integrated data from CTMS/RINS have enabled near-real-time assessment of patient accrual and accelerated reimbursement from industry sponsors. For institutions with bioinformatics or programming capacity, the CTMS/RINS integration provides a powerful model for tracking and improving clinical trial efficiency, compliance, and cost-effectiveness.

## Introduction

Patient accrual has been identified as a key metric for gauging the efficiency of a clinical trial by the Clinical and Translational Science Awards (CTSA) consortium [1,2], which is intended to be a national laboratory for testing process improvements for clinical research [3]. Low-accruing clinical trials present a double hurdle to translating research breakthroughs to the clinic. First, because they do not meet recruitment goals, they are unlikely to reach the statistical power to be clinically relevant and as such raise ethical questions about whether it was appropriate to enroll participants in a trial that provides no insight [4-12]. Second, they typically end up costing their host institutions money, potentially souring leadership on the clinical research enterprise [5,6,10,13,14].

Tracking accrual more closely could identify impediments [15] and enable CTSA hubs to intervene early in underperforming trials. Such early intervention could increase the chances that trials will be clinically meaningful and financially viable by helping study teams reach accrual targets and other trial milestones on which industry sponsors condition payment. However, accrual and trial financials have been difficult to track and benchmark because the related data are often siloed in disparate systems that do not communicate [1,16,17].

In September 2020, the South Carolina Clinical and Translational Research (SCTR) Institute, a CTSA hub with a home at the Medical University of South Carolina (MUSC), addressed the problem of data fragmentation by implementing a clinical trial management system (CTMS) and integrating it into its in-house Research Integrated Network of Systems (RINS) [18]. The CTMS provides access to patient-level data, while RINS tracks study-level data across disparate systems using a unique study identifier, the Research Master ID (RMID), and application programming interfaces (APIs).

The CTMS/RINS integration enables SCTR to monitor regulatory and recruitment milestones, information that is in high demand by industry sponsors [19]. It also aids SCTR, the Office of Clinical Research (OCR), and study teams in tracking invoicing to and reimbursement from industry sponsors to improve the financial performance of trials. Finally, it allows SCTR to identify and intervene in low-accruing trials, make data-driven decisions about which trials are the best fit for MUSC’s patient population and clinical specialties, and set realistic recruitment goals. CTMS/RINS is a powerful systems integration model developed to improve clinical trial quality, efficiency, and cost-effectiveness and could prove useful to other CTSA hubs or academic medical centers that are willing to invest in the programming capacity needed to tailor it to their own digital ecosystems.

## Methods

### Project Inception

In the past 10 years, SCTR and the Biomedical Informatics Center (BMIC), recognizing the importance of reliable, robust data to the continuous process improvement of clinical trials, have taken steps to address the barrier of data fragmentation. They co-developed SPARCRequest® [20,21], an open-source research transaction management system, and interfaced it with the electronic health record (EHR) to support research billing compliance. They created and mandated a unique RMID for each study so that study-level data could be tracked across disparate systems, built the APIs to create RINS and a research data mart to extract the integrated data, and developed performance dashboards for tracking key metrics. In addition to SPARCRequest, RINS provides access to data from the EHR, the electronic institutional review board (eIRB), and financial, sponsored awards, and faculty management systems (Supplemental Table).

Although these innovations enabled robust monitoring of protocol-level data, they did not provide access to the patient-level data needed to track clinical trial participant accruals and associated financials. To address that gap, SCTR leadership applied for and in 2019 was awarded an administrative supplement from the National Center for Advancing Clinical Trials to procure a CTMS and implement it enterprise-wide as a cornerstone of the RINS platform. In March 2019, MUSC hosted a liftoff meeting attended by representatives from several CTSA and clinical and translational research hubs to explore best practices for deploying an enterprise-wide CTMS and integrating data for metric tracking.

### Workflow Mapping and Initiation of Data Migration

The core OnCore support team (an associate director, a trainer, a business analyst, and a program coordinator) collaborated with the vendor, project stakeholders, BMIC’s Ruby on Rails web development team, infrastructure, and EHR research teams as well as SCTR, OCR, and HCC subject matter experts (SMEs) to customize integrations and workflows between the CTMS, SPARCRequest, and the EHR. These integrations eliminated duplicative data entry, ensured data and workflow harmonization across systems, and reduced administrative burden for data entry and cleanup.

With the goals of transitioning cancer trial data smoothly from the legacy system used for cancer trials (Velos) to the new enterprise-wide CTMS (OnCore) and supporting National Cancer Institute protocol review and reporting requirements, the core team mapped Hollings Cancer Center (HCC) clinical trial workflows and required data points. System analysts and SMEs met with vendor analysts to determine the migration schedules for the transition, and group administrators for HCC then tested and validated the migrations before the previous CTMS was taken offline. Essential data from the legacy system were preserved in the research data mart for record-keeping purposes. A workflow for studies outside HCC was also created and, where appropriate, aligned with that of the HCC to create institutional workflow harmonization.

Key to the implementation planning process was identification of best practices for integrating data to support tracking and evaluation of common metrics. Stakeholders opted to integrate the CTMS into the existing RINS architecture by introducing the RMID, the unique research study identifier at MUSC, into the CTMS, using an available protocol-specific text field in the CTMS’ API. The RMID could then be used to link CTMS records back to the rest of the records in the institution’s research data mart, where data relevant to research metrics from the CTMS and other linked systems would be extracted nightly using Extract, Transform, Load (ETL) tools. As a second linking key, the “Protocol No.” in OnCore is also mapped to its corresponding Protocol ID in SPARCRequest. These customizations enabled the CTMS-enhanced RINS platform to support key institutional research metric tracking, process improvement, and reporting needs.

### Application Programming Interface Design and Testing

**Fig. 1.**
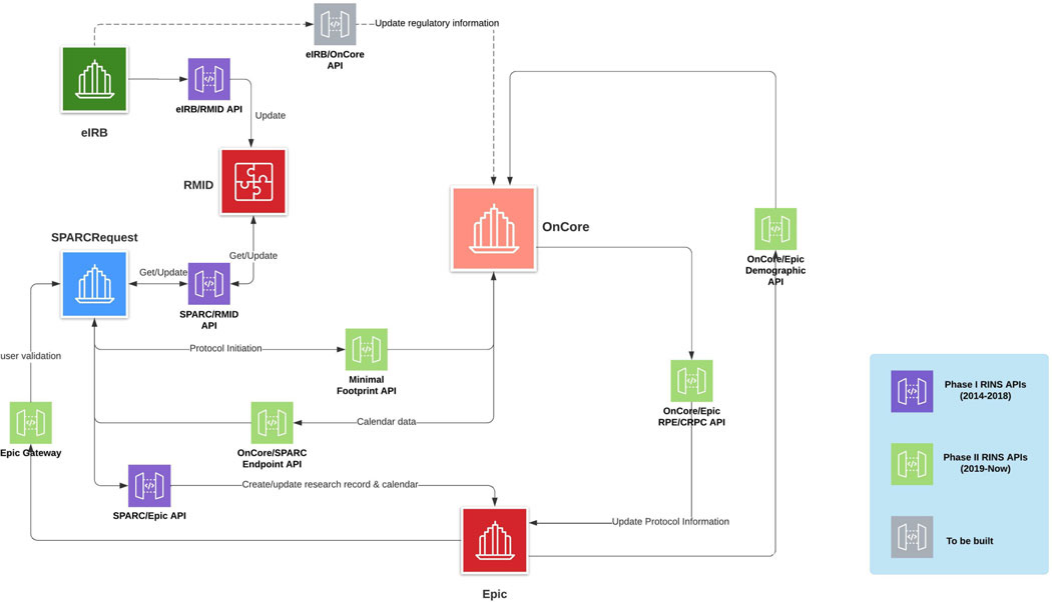
Diagram of the application programming interfaces (APIs) developed for the Research Integrated Network of Systems (RINS) and clinical trial management system integration (CTMS). Epic is the electronic health record system and OnCore the enterprise-level CTMS used by the Medical University of South Carolina. CRPC = Clinical Research Process Content; eIRB = electronic institutional review board; RMID = Research Master ID; RPE = retrieve process for execution; SPARCRequest® = Services, Pricing, & Application for Research Centers.

Having learned the capabilities of the selected CTMS, the core team compared institutional needs and existing SPARCRequest/EHR API functionalities and decided to leverage the merits of both platforms. The CTMS has two native Retrieve Process for Execution (RPE) interfaces with the EHR: one of them pushes protocol information (RPE push) and then Calendar information (CRPC push) into the EHR, and the other one pulls patient demographic information into the CTMS from the EHR and sends updates regarding patient recruitment status back to the EHR (Fig. 1). Both interfaces were implemented at MUSC after extensive configuration and testing. The decision was also made to implement the CTMS’s optional receiving API to preserve MUSC’s SPARCRequest/EHR innovations and optimized workflows, reduce duplicative systems builds, and stimulate utilization of the CTMS. The SPARCRequest team designed and built a custom JavaScript Object Notation (JSON) message that is triggered from SPARC Dashboard by designated interface users. This message creates a new protocol in the CTMS with the minimal footprint fields identified.

Other MUSC adaptations included the design of a new “calendar recipient” (Endpoint) API for SPARCRequest that enabled it to receive study calendars from the CTMS (Fig. 1). This adaptation made possible the continued use of the API between SPARCRequest and MUSC’s EHR and allowed more flexibility for future SPARC development tailored to MUSC. Thanks to the new Endpoint API, SPARCRequest receives calendar structure from the CTMS as studies are created to ensure consistency across systems and then sends the calendar on to the EHR. Routing the calendars through SPARCRequest ensures that all clinical and clinical research services use the same calendar and key protocol record fields. SPARCRequest can also link related services in its catalog to support optimization of research workflows, as for example linking research billing compliance review with ClinicalTrials.gov review.

### Phased Rollout

A phased approach was used for the CTMS rollout, beginning with the migration of studies from the legacy HCC system in September 2020. Because implementation occurred during the COVID-19 outbreak, the OCR budgeting and invoicing team, which oversees the financial performance of industry-supported trials at MUSC, were the first to pilot the new CTMS for COVID vaccine trials, followed by SCTR’s Research Coordination and Management and study teams in other interested departments. HCC was fully onboarded on May 19, 2021.

## Results

### Uptake of New CTMS/RINS Integration

In the year since the implementation of the CTMS and its integration with RINS (September 29, 2020 to September 30, 2021), HCC, SCTR, and the OCR have used it to support their portfolio of research studies with high satisfaction. A total of 601 protocols have been pushed from SPARCRequest to the CTMS through the minimal footprint API, 216 calendars have been imported from the CTMS back into SPARCRequest, and 296 protocols have been pushed from the CTMS to the EHR through the RPE interface. The recruitment status of the 2,602 participants who have been enrolled in trials has been updated from the CTMS to the EHR a total of 4,055 times.

**Fig. 2.**
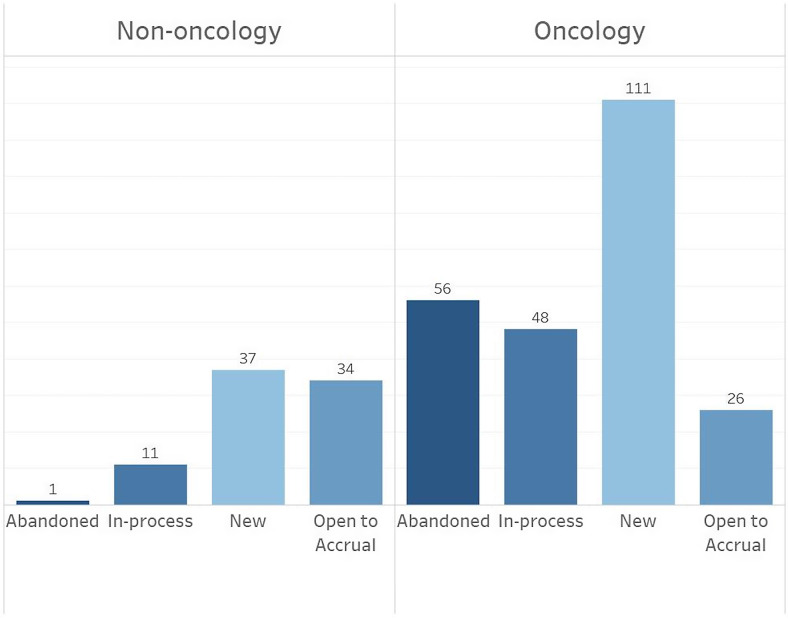
The number of oncology and non-oncology protocols in the clinical trial management system, with status updates, during the first year of implementation (*N* = 324).

Of the 601 protocols pushed from SPARCRequest to the CTMS, 324 (53.9%) have had status updates made by the study team, indicating whether the trial is new, in process of being activated, open to accrual, or abandoned (Fig. 2). Although a minimal footprint record exists in the CTMS for the remaining 275 trials (45.8%), study teams have not yet completed the necessary additional steps to activate those trials fully and thereby enable tracking.

### Improved Ability to Track Patient Accrual

**Fig. 3.**
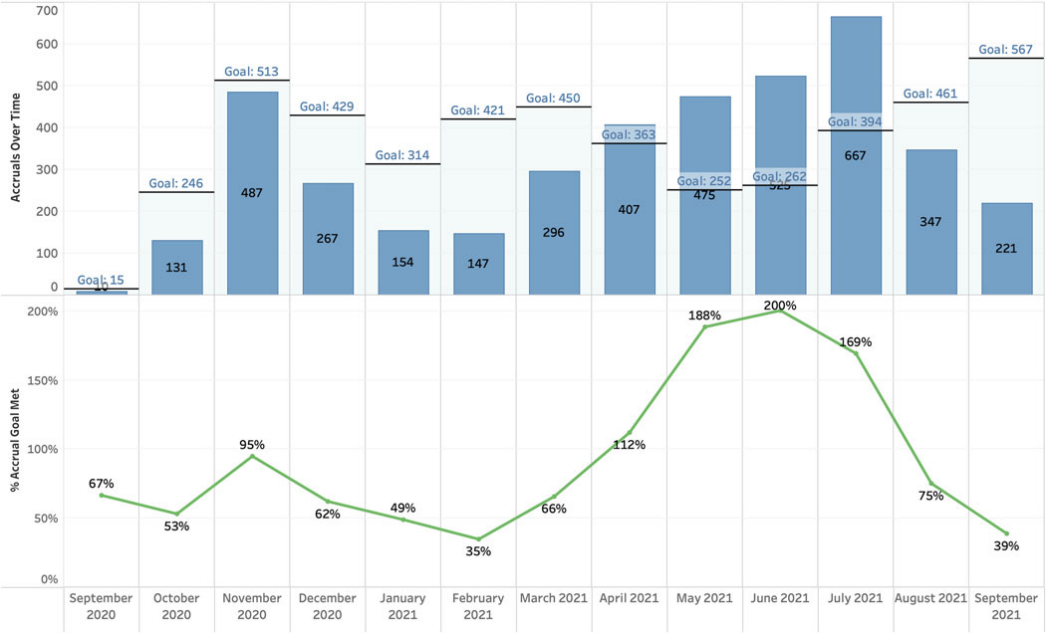
Target and actual accruals over time (September 29, 2020 – September 30, 2021*) for protocols with goals and duration in the clinical trial management system (OnCore). *The accrual date/month is based on when study teams updated a participant’s status to “on study.”

The CTMS/RINS integration offers access to near-real-time data from all linked systems and consistent definitions of data points (e.g., study start date), enabling SCTR and institutional leadership to track patient accrual for studies using the CTMS. Accrual reports can be provided at the individual study or departmental level or for the entire portfolio of studies entered into the CTMS. Principal investigators, department chairs, and clinical research leadership can use these reports to compare actual accrual against target enrollment to assess the progress of trials and to set more realistic recruitment goals for future trials (Fig. 3).

### Improved Ability to Track Financials and Recover Funds from Sponsors

**Fig. 4.**
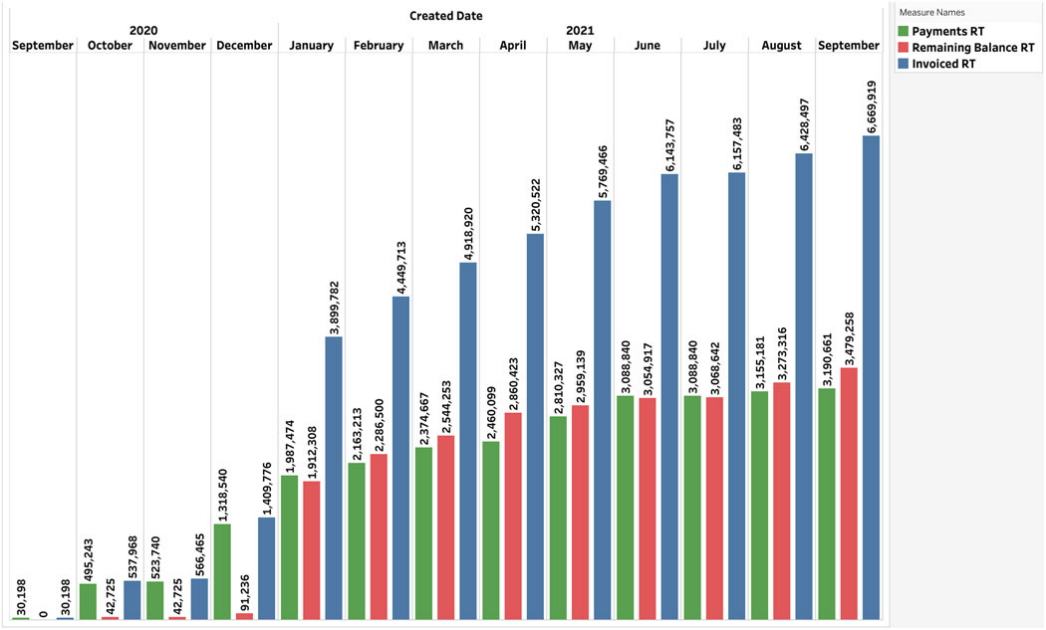
Invoices, reimbursements, and remaining balances, by month, for 75 industry-sponsored trials (September 2020−September 2021). *RT = running total.

The CTMS/RINS integration allows for much more granular tracking of earned revenue, invoices, and payments received than was possible with RINS alone. These data are extracted into a data mart nightly and can then be accessed through business intelligence dashboards and reports (i.e., Tableau at MUSC), which graphically represent earned revenue, both received and outstanding, in near-real-time (Fig. 4). These dynamic reports can be broken down by department, principal investigator, sponsor, clinical specialty, or disease type, enabling department chairs, principal investigators, and study teams to monitor and take action to improve the financial health of their studies. For example, the “OCR Invoicing Phase Report” dashboard in Tableau, which has been shared with study teams, is being used to track the budgeting service status and the reconciliation of the sponsor invoicing items by date from the financial system. The CTMS/RINS integration also enables SCTR to track and invoice for the clinical research services that it provides. Study teams can also improve the return on investment for future studies by comparing their pre-negotiation assessments of the study’s budget, housed in SPARCRequest, with actual costs. In short, the CTMS/RINS integration provides study teams and CTSAs with a uniquely powerful tool for monitoring a study’s financial performance.

### Early Success

The OCR budgeting/invoicing team, an early adopter of the CTMS/RINS integration, used the platform to facilitate the prompt activation of and recruitment for two COVID-19 vaccine trials, which randomized 724 participants between August 28, 2020 and March 31, 2021. Taking on large trials so quickly could have had negative financial consequences for MUSC had they not been run efficiently, but CTMS/RINS provided study teams and SCTR and OCR staff the enrollment, billing, and reporting tools they needed to track recruitment-conditional invoices and their reimbursement, ensuring their financial viability. CTMS/RINS has supported $6.1 million in invoicing for these high-volume, resource-intensive trials.

Although this early success is very promising, the full potential of CTMS/RINS will be realized only once all clinical research studies have been onboarded, providing a complete data set to support metric tracking, continuous quality improvement, and reporting. The challenge for CTMS adoption is to achieve leadership and study team buy-in by proving value versus workload.

## Discussion

Early attempts to leverage technology to improve the efficiency of clinical trials focused on standalone systems for discrete processes. While some of these standalone systems were very well suited to the particular tasks for which they were designed, they did not integrate with one other [11,22,23], leading to a fragmented clinical trial infrastructure [24]. Such fragmentation was identified as a serious hurdle to attaining the data needed to calculate the proposed CTSA accrual metric [1,25,26]. Similar to MUSC’s experience, other hubs had to access the required data from paper records or individually contact the study principal investigators. Hubs with electronic databases often had to access information from a variety of standalone systems, slowing the process and creating the risk of duplicative files [26,27]. Even hubs with a CTMS struggled to report on elements of the metric if there was no appropriate field in the CTMS. Although the common accrual metric initiative is currently paused due to the pandemic and the need to assess barriers to data collection at CTSA hubs [28], the CTSA consortium continues to recognize that it is imperative to address the problem of uninformative trials through efficient management of trial portfolios and interventions on low-enrolling trials [29].

For MUSC, CTMS/RINS has provided a relatively simple means – the addition of an RMID unique identifier – to link systems that host data needed to track clinical trial efficiency, accrual, and cost-effectiveness across disparate systems, without the need for radical reprogramming.

### Streamlining Accrual Tracking

Prior to the CTMS/RINS integration, tracking clinical trial milestones such as recruitment required SCTR staff to contact study teams directly for the necessary data, presenting an overwhelming operational burden. Data were sometimes inaccurate or inconsistent due to time lags, staff turnover, and differences in how study teams and siloed systems tracked information. Collecting the data was time-consuming, if not impossible, and did not yield timely or automatically updated information for reporting.

The CTMS/RINS integration allows SCTR to track accrual of all participating studies in near-real-time. APIs enable bidirectional exchange of information between the EHR, SPARCRequest, and the CTMS, and integrated data from all linked systems are available via the research data mart. SCTR leadership can use these data to identify low-performing trials early and intervene to get them back on track. Business intelligence dashboards provide snapshots of progress toward accrual goals for individual trials and for all trials in the CTMS. As adoption of the CTMS grows to include most or all of the trials at MUSC, these dashboards will be invaluable not only for monitoring current trials but also for informing future decisions about which trials to select and what enrollment targets to set.

### Centralizing Financial Reporting

Institutions are unlikely to support a clinical research enterprise that is bad for their bottom line, and industry sponsors are unlikely to choose sites that cannot document a proven accrual track record. Academic medical centers can bill insurance for standard-of-care procedures performed during a trial but still need to recoup monies from sponsors for research-specific tasks that lie outside of usual care, and study teams require reimbursements for research personnel and service costs. Industry sponsors often condition payment on hitting accrual or other study milestones, and so the ability to track attainment of those milestones is critical to recovering more money due from sponsors and ensuring the financial viability of a trial.

Prior to CTMS implementation, MUSC had no centralized way to track clinical trial contractual earned revenue, much less match it with revenue received from industry sponsors. Moreover, missed revenue from invoiceable items, such as start-up and close-out costs and participant procedures, were sometimes not recovered at all, resulting in a significant loss to the institution. The access to both patient- and protocol-level data made possible by the CTMS/RINS integration provides study teams and SCTR with a robust means of tracking accrual-conditional reimbursement as well as study-specific costs to ensure appropriate reimbursement by sponsors.

### Right-Sizing Trials

The ability to closely track financial and accrual performance of past trials using CTMS/RINS will also enable clinical research leadership to select trials that are a better fit for the institution and to set realistic expectations for enrollment and financial recovery. Inexperienced clinical researchers can sometimes inadvertently inflate the enrollment targets for their trials, not realizing they will not have access to patient populations of that size [9]. Overinflating the enrollment target and the award from the sponsor can set unrealistic expectations. For example, if a researcher aims to enroll 20 patients but enrolls only five, MUSC receives funds for only five and appears to under-enroll. Right-sizing trials may seem to lower the industry award amount but in fact more accurately projects anticipated revenue and recruitment goals, leading to improved trial efficiency.

### Challenges

Historically, adoption has been a challenge for CTMS implementations, in part because staff can be reluctant to learn yet another new system [30]. The SPARCRequest/CTMS API eased the burden on staff by pushing a minimal footprint record for each trial in SPARCRequest to the CTMS, resulting in approximately 54% of study teams fully activating their trials. The integration also reduced the burden on study teams and improved the consistency of data by eliminating the need for duplicative entry in disparate systems. For instance, study calendars, which are shared between SPARCRequest, the CTMS, and the EHR, need only be built in one system and will auto-populate in the rest. Such real-time access to the same scheduling information enables both clinical and research teams to track a study’s enrollment and other progress more easily, document study milestones for sponsors, and stay on top of invoices and their reimbursement.

However, despite these integrations intended to ease the burden on staff, uptake of the CTMS has been slower than expected. COVID has had a significant impact on the CTMS rollout, requiring recalibration of timelines and expectations as study teams coped with the pandemic. As the pandemic neared its two-year mark, SCTR leadership made the decision to mandate the usage of the CTMS for all new qualifying clinical trials beginning March 1, 2022. It is hoped that the mandate will smooth the next phase of CTMS implementation.

Other challenges that provide future development opportunities include study amendments and APIs. A calendar can only be pushed to SPARCRequest from the CTMS once, where it serves as the blueprint for the workflow in the EHR. Study amendments require manual manipulation to harmonize calendars in the CTMS and SPARCRequest, and a more automated way to address this obstacle is needed.

SPARCRequest and its surrounding APIs, essential components of RINS, are open source, but tailored to systems used in MUSC’s digital ecosystem. However, institutions with programming capacity can tailor the tool to their own needs and digital ecosystems using this CTMS/RINS model as a roadmap/blueprint for how to integrate protocol- and patient-level data to track clinical trial efficiency.

### Advice to Development Teams

Good API design takes into account stakeholder requirements and workflows. We used both data flow visuals and workflow mapping overlay visuals to achieve agreement among the user groups before diving into the coding. Involving the main API developer early on, as soon as the system data flow was designed, has enabled us to adapt to the rapid rollout. The CTMS integration team’s involvement was also crucial for everything from retrieving the correct API documentation to configuration of the CTMS webhooks, to testing and revisions.

## Future Directions

Future directions include the continuous enrichment of RINS with additional data sources and interconnections that support and advance the science of translational research. An eIRB/CTMS API was deployed in February 2021 and will allow for automatic entry of regulatory information into the CTMS, tracking of IRB approval turnaround time, improved reporting compliance, and enhanced data harmonization across systems. Robust data on study activation timelines, accrual, and financial performance will support continuous process improvement of the quality and efficiency of clinical trials, ultimately bringing new therapies to patients faster, a CTSA goal.

The core team will also develop and disseminate to the SPARC OS community and other interested institutions a best practices model for the CTMS/SPARC/EHR interface and RMID data synchronization logic, along with eIRB/RMID and SPARC/RMID APIs.

## Conclusion

The CTMS/RINS integration has allowed for more robust tracking of study milestones and financial performance by providing access to both patient- and study-level data housed in all relevant clinical, research, and financial systems. The platform has enabled near-real-time tracking of accrual (e.g., current enrollment vs. recruitment goals) and financial performance (amount invoiced vs. reimbursed), data that are crucial to continuous process improvement initiatives. For institutions willing to invest in the programming capacity to tailor CTMS/RINS to their digital ecosystems, it could provide an effective model for monitoring clinical trial efficiency and cost-effectiveness with minimal changes to existing clinical trial systems and processes.

## Supplementary material

For supplementary material accompanying this paper visit https://doi.org/10.1017/cts.2022.382. click here to view supplementary material
