# Supplementary material for: An integrated approach to improve clinical trial efficiency: Linking a clinical trial management system into the Research Integrated Network of Systems
Source: J Clin Transl Sci. 2022 Apr 1;6(1):e63. doi: 10.1017/cts.2022.382 (PMC9161043; doi:10.1017/cts.2022.382)
Supplement: Supplementary file 1 [file S205986612200382Xsup001.docx]

**Supplemental Table.** Research systems and data exchanges involved in the CTMS/RINS integration*****

| **System** | **Purpose** | **Primary stakeholders** | **Source-of-truth data fields**** | **Data types consuming from another primary system**** |
| --- | --- | --- | --- | --- |
| Research Master ID | Unique Identifier Ticketing | Institutional research community | RMID | Protocol titles; Principal investigator; Pro #; IRB state; SPARC ID  Cayuse Award ID |
| eIRB | Electronic institutional review board | Institutional compliance administrators and review board | Protocol titles; IRB of record (i.e. Pro #);  Principal investigator  Type of IRB;IRB status; Date of IRB approval; Planned enrollment | RMID |
| SPARC- Request | Research transaction management | CTSA and institutional administrators and researchers | SPARC ID  CTSA service involvement  OCR involvement  Sponsor name | RMID  Protocol titles  Principal investigator  Pro #  IRB status |
| EPIC | Electronic health record | Clinicians and clinical researchers | Patient registry  Recruitment start date***  Last patient enrollment date***  Enrollment counts*** | SPARC ID  Protocol titles  Principal investigator  Study calendar  Participant enrollment status |
| Cayuse | Grants and contracts | Grants and contracts administrators | Sponsor protocol #  Award #  Award title  Award amount | RMID |
| Clincard | Participant remuneration | Clinical trials financial office | Participant remuneration  Cards distribution  Tax information | RMID  Pro #  SPARC ID |
| Smart-Stream | Financial | Clinical trials financial office | Award received amount  Expenditures | Award # |
| OnCore | Clinical trials management | Clinical trials study teams and administrators | Study calendar  Participant enrollment status | SPARC ID  Protocol titles  RMID ****  Patient registry |

***** This Table lists the research systems already in use at the Medical University of South Carolina before the implementation of an enterprise-wide clinical trial management system (CTMS) and its integration with the Research Integrated Network of Systems (RINS), along with the source-of-truth data fields and data adoption from other primary systems for data consistency. The last row also details the vision for the role that the CTMS will play in this network of systems, and some of the source-of-truth records' transitioning plans. Among these systems, the SPARCRequest® (<https://github.com/sparc-request/sparc-request>) and SPARCFulfillment applications (<https://github.com/sparc-request/sparc-fulfillment>) and the ​​​​Research Master Identifier (RMID) (<https://github.com/sparc-request/research-master-id>), have all been released as open-source software on Github with a BSD3 license. CTSA = Clinical and Translational Science Awards; eIRB = electronic institutional review board; OCR = Office of Clinical Research; SPARCRequest = Services, Pricing, & Application for Research Centers.

****** This table lists some but not all key data fields.

*** These fields could only be reported from EPIC before the implementation of the CTMS; as the CTMS is being mandated across the institution, the source-of-truth will shift.

**** A feature undergoing development.
